# ABA-Dependent Salt Stress Tolerance Attenuates *Botrytis* Immunity in Arabidopsis

**DOI:** 10.3389/fpls.2020.594827

**Published:** 2020-11-17

**Authors:** Eva Haller, Tim Iven, Ivo Feussner, Mark Stahl, Katja Fröhlich, Birgit Löffelhardt, Andrea A. Gust, Thorsten Nürnberger

**Affiliations:** ^1^Department of Plant Biochemistry, Center for Plant Molecular Biology, Eberhard Karls University of Tübingen, Tübingen, Germany; ^2^Department of Plant Biochemistry, Göttingen Center for Molecular Biosciences (GZMB), Albrecht von Haller Institute, University of Göttingen, Göttingen, Germany; ^3^Analytics Unit, Center for Plant Molecular Biology, Eberhard Karls University of Tübingen, Tübingen, Germany; ^4^Department of Biochemistry, University of Johannesburg, Johannesburg, South Africa

**Keywords:** Arabidopsis thaliana, combinatorial stress, salt stress, immunity, Botrytis cinerea, Pseudomonas syringae, abscisic acid

## Abstract

Plants have evolved adaptive measures to cope with abiotic and biotic challenges simultaneously. Combinatorial stress responses require environmental signal integration and response prioritization to balance stress adaptation and growth. We have investigated the impact of salt, an important environmental factor in arid regions, on the *Arabidopsis* innate immune response. Activation of a classical salt stress response resulted in increased susceptibility to infection with hemibiotrophic *Pseudomonas syringae* or necrotrophic *Alternaria brassicicola*, and *Botrytis cinerea*, respectively. Surprisingly, pattern-triggered immunity (PTI)-associated responses were largely unaffected upon salt pre-treatment. However, we further observed a strong increase in phytohormone levels. Particularly, abscisic acid (ABA) levels were already elevated before pathogen infection, and application of exogenous ABA substituted for salt-watering in increasing *Arabidopsis* susceptibility toward *B. cinerea* infection. We propose a regulatory role of ABA in attenuating *Botrytis* immunity in this plant under salt stress conditions.

## Introduction

Perception, response and adaptation to an ever-changing environment are fundamental processes that occur in all organisms. For plants as sessile organisms it is crucial to be able to integrate information from environmental cues and to mount corresponding cellular responses in case of harmful conditions. Salinity is one of the major abiotic factors limiting agronomic productivity and is thus a primary cause of crop losses worldwide. Approximately one fifth of the total land mass and nearly one half of all irrigated land are affected by salinity (Mahajan and Tuteja, 2005). Salinity has a profound impact on plant physiology (Julkowska and Testerink, 2015; Yang and Guo, 2018). By disrupting the ionic and osmotic equilibrium of the cell, salinity causes hyperosmotic stress and oxidative damage in plants (Yang and Guo, 2018; van Zelm et al., 2020), which can lead to plant weakening and reduced growth and even plant death (Julkowska and Testerink, 2015). To cope with high salinity, plants have evolved several mechanisms, such as a selective ion uptake/exclusion, the compartmentalization of toxic ions, the adjustment of photosynthetic and energy metabolism or the accumulation of antioxidative enzymes and the fine-tuning of phytohormone levels (Munns and Tester, 2008; van Zelm et al., 2020).

Another important component of the environmental sensing network of a plant is the innate immune system that prevents the majority of microbes from infecting tissues. The plant innate immune system comprises membrane-resident pattern recognition receptors (PRRs), which recognize danger signals that can be derived from the plant itself (danger-associated molecular patterns, DAMPs) or can constitute invariable, conserved microbial structures (microbe-associated molecular patterns, MAMPs) (Couto and Zipfel, 2016; Gust et al., 2017; Saijo et al., 2018; van der Burgh and Joosten, 2019; Albert et al., 2020). Upon MAMP perception a multilayered plant immune response is mounted, including the activation of mitogen-activated protein kinase (MAPK) cascades, the accumulation of reactive oxygen species (ROS), the production of defense-associated phytohormones, and transcriptional reprogramming (Yu et al., 2017; Saijo et al., 2018). An effective immune response will thus ultimately prevent further pathogen proliferation and spread. Only pathogens that can circumvent recognition or suppress host defenses can establish successful infections and cause disease (Toruno et al., 2016; Miwa and Okazaki, 2017).

In nature, one stress factor often occurs simultaneously with other stresses. For instance, salinity is often found in combination with drought in arid areas (Bartels and Sunkar, 2005). Indeed, during the last years, not only tolerance mechanisms to individual stresses such as salinity have been studied and underlying molecular mechanism have been characterized (Yang and Guo, 2018), but also an increasing number of studies described the performance of a given plant to combinatorial stresses (Mittler, 2006; Suzuki et al., 2014; Zhang and Sonnewald, 2017). Many reports have been dealing with combinations of two different abiotic stresses in crop plants such as drought and heat stress (Mittler, 2006). More recently, abiotic factors have been described to modulate performance and efficacy of the plant immune system (Suzuki et al., 2014; Zhang and Sonnewald, 2017; Velasquez et al., 2018; Saijo and Loo, 2020). For instance, tomato and *Arabidopsis* grown under high salinity conditions displayed an increased susceptibility to infection with obligate biotrophic oomycete *Phytophthora spp* (Dileo et al., 2010; Bostock et al., 2014), whereas tomato resistance toward infection with the necrotrophic fungus *Botrytis cinerea* was not affected under salt stress (Achuo et al., 2006). Conversely, drought-stressed tomato are less susceptible to *Botrytis* infection (Achuo et al., 2006), whereas drought stress in *Arabidopsis* plants resulted in an increased *Botrytis* growth rate in an accession-dependent manner (Coolen et al., 2019). Apparently, the effect of a given abiotic stress on subsequent pathogen infections depends on the plant species and the combination of stresses under investigation. Heat stress also affects *Arabidopsis* immunity, and it was shown that both virulent and avirulent *Pseudomonas syringae* pv. *tomato* strains can grow better in heat-stressed plants (Wang et al., 2009).

Here, we aimed at investigating the performance of salt-stressed *Arabidopsis* plants during biotic interactions. We observed that salt pre-treated *Arabidopsis* plants are impaired in their immune response toward both necrotrophic fungal and biotrophic bacterial pathogens. Surprisingly, activation of PTI-associated plant defenses was not altered upon such treatment. We have further found substantial alterations in various plant hormone levels upon combined stress application and describe a role of abscisic acid as disease-promoting factor in salt-stressed plants.

## Materials and Methods

### Plant Material and Salt Treatment

*Arabidopsis thaliana* ecotype Columbia-0 (Col-0) was cultivated on soil as described (Brock et al., 2010). Salt stress was applied to 5 weeks old *Arabidopsis* plants grown on soil in 100 ml pots by watering plants for 4 days with 25 ml of a 150 mM NaCl solution. Subsequently, pathogen infections or MAMP treatments were performed.

### Pathogen Cultivation and Infection

Infection assays with *Pto* DC3000 and the fungi *Alternaria brassicicola* isolate MUCL 20297 or *Botrytis cinerea* isolate BO5-10 were performed as described previously (Kemmerling et al., 2007) on 4–5 weeks old plants grown on soil in environmental chambers for 4–5 weeks under short-day conditions (8 h photoperiod, 22°C, 40–60% humidity) at a light intensity of 150 μmol/m^2^ s.

Histochemical analysis of plant cell death and fungal growth by trypan blue staining and of H_2_O_2_ production by staining with 3,3′-diaminobenzidine tetrahydrochloride (DAB) was performed as described (Torres et al., 2005). Leaves were analyzed by light microscopy.

For fungal DNA quantification after *A. brassicicola* or *B. cinerea* inoculation, 3 infected leaves of six different plants were pooled and frozen in liquid nitrogen. Total DNA was isolated using a modified method after Edwards et al. (1991). Briefly, 200 mg leaf tissue was homogenized in Edwards buffer (200 mM Tris/HCl, pH 7.5; 250 mM NaCl; 25 mM EDTA, pH 8.0; 0.5% SDS), followed by an extraction with phenol/chloroform/isoamylalcohol (25:24:1) and precipitation with 1 volume isopropanol. Fungal biomass was determined by RT-qPCR using the SYBR Green qPCR Master Mix (Thermo Fisher Scientific). The relative concentration of genomic DNA levels of the *Alternaria* 5.8S ribosomal RNA gene or the *Botrytis Actin* gene to *Arabidopsis Rubisco* (large subunit) levels was used to quantify fungal biomass (Fradin et al., 2011). Specific primers are listed in Supplementary Table S1.

Chlorophyll content in at least eight leaves per treatment was measured photometrically in methanol extracts as described (Porra et al., 1989).

### Immune Assays and RT-qPCR Analyses

For the detection of activated MAPKs, immunoblot analyses using the anti-phospho p44/42 MAP kinase antibody (Cell Signaling Technology) were performed as described (Zhang et al., 2013). The detection of ROS in Arabidopsis leaf pieces was performed as described (Wan et al., 2019). Callose deposition in *Arabidopsis* leaves was stained with aniline blue 24 h after MAMP infiltration as described (Gomez-Gomez et al., 1999). Digital pictures of stained leaves were taken under UV-light with the microscope and callose depositions were quantified by counting light, fluorescent pixels with the Adobe Photoshop CS “select range” tool. Per treatment at least 10 pictures of at least 5 different leaves were analyzed.

For transcript profiling, RNA isolation and RT-qPCR analysis of plant material were performed as described previously (Zhang et al., 2013). The sequences of the primers used for PCR amplifications are listed in Supplementary Table S1.

### Phytohormone Analyses

The accumulation of salicylic acid, jasmonic acid-isoleucine, abscisic acid and indole-3-acetic acid in *Arabidopsis* leaf tissue was determined by HPLC-MS-MS as previously described (Iven et al., 2012). Ethylene accumulation was determined by gas chromatography as described (Wan et al., 2019).

## Statistical Analysis

For statistical analysis of data based on Student’s *t*-tests, calculations were performed on a minimum of three independent data sets, assuming two-sample equal variance and a two-tailed distribution. Normal distribution data sets were evaluated using the *post-hoc* comparisons following one-way ANOVA (Dunn test) multiple comparison analysis at a probability level of *p* < 0.05. All statistical analyses were carried out with SAS jmp.

## Results

### Salt-Treatment Impairs Growth of Arabidopsis Plants

The model plant *Arabidopsis thaliana*, similar to most crop plants, falls into the class of salt-sensitive plants, called glycophytes (Byrt and Munns, 2008; Flowers and Colmer, 2008; Katori et al., 2010). For such plants, saline soils impose considerable stress affecting growth, development and physiology (Yang and Guo, 2018). We first conducted control experiments in order to verify the effect of salt treatment on *Arabidopsis* plants under our culture conditions. Within 4 days of applying an aqueous solution of 150 mM sodium chloride to the soil of 4–5 weeks old plants, we observed transcript accumulation of the two typical salt-responsive genes *SOS1* and *DREB1a* (Supplementary Figures S1A,B). Macroscopically, plants salt-stressed for 4 days displayed growth retardation when compared to regularly watered plants (Supplementary Figure S2A). Total fresh weight and amount of leaves per rosette did not change, but individual leaves of salted plants were shorter and appeared darker green than control plants (Supplementary Figures S2B–E). Notably, total chlorophyll contents were not altered in salt-treated plants (Supplementary Figure S2F). Likewise, salt stress did not result in macroscopic symptoms, such as wilting (Supplementary Figure S2A), or in altered expression of senescence-associated gene *YLS4* (Supplementary Figure S1C), indicating that salt-induced stunted plant growth is not accompanied by the induction of premature senescence.

### Salt Treatment Prior to Infection Results in Decreased Plant Resistance Toward Both, Hemibiotrophic and Necrotrophic Pathogens

**FIGURE 1:**
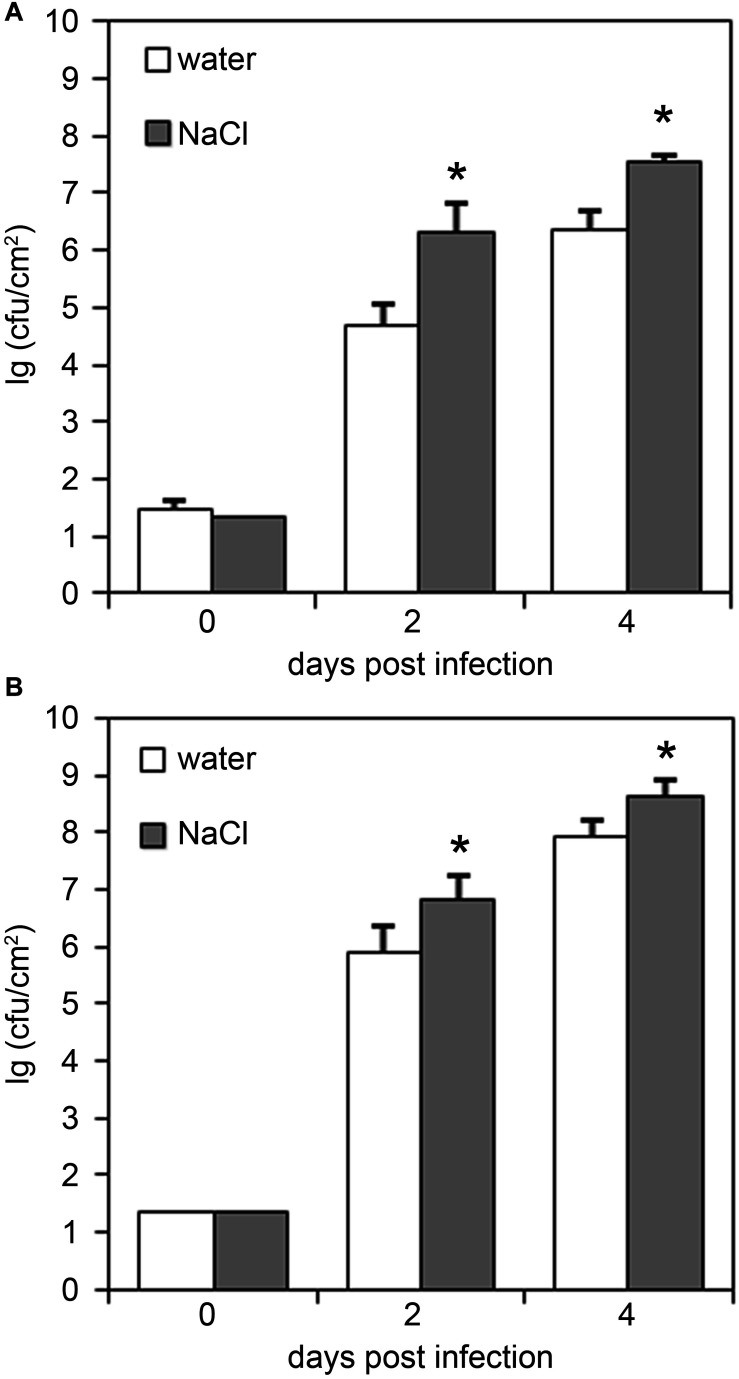
Plants pre-treated with salt are impaired in their resistance to bacterial infection. Five-weeks-old plants were either normally watered or treated with a 150 mM NaCl solution for 4 days before infection with either **(A)** *Pto* DC3000 or **(B)** *Pto* DC3000(*AvrRPM1*). Leaves were infiltrated with 10^4^ colony forming units ml^− 1^ (cfu/ml) and bacterial growth was monitored at 2 or 4 days post infiltration. Data represent means ± SD of six replicate measurements per time point, significant differences to the water-treated controls are indicated by an asterisk (*p < 0.05; Student’s *t*-test). Representative data of at least four independent experiments per bacterial strain are shown.

To study the impact of salt stress on the performance of the plant immune system, we next infected salt-stressed plants with the hemibiotrophic bacterium *Pseudomonas syringae* pv. *tomato* (*Pto*). Previous studies reported that salt- and drought-treatment reduced the resistance of *Arabidopsis* plants to subsequent infection with the disarmed strain *Pto* DC3000 hrcC^–^ (Berens et al., 2019). We observed that infection with both the virulent strain *Pto* DC3000 or the avirulent strain *Pto* DC3000(*AvrRpm1*) resulted in bacterial growth rates that were significantly enhanced in salt-pre-treated plants relative to those observed in control plants (Figure 1). Hence, salt-pre-treatment resulted in an enhanced susceptibility of *Arabidopsis* plants toward infection with hemibiotrophic bacteria.

**FIGURE 2:**
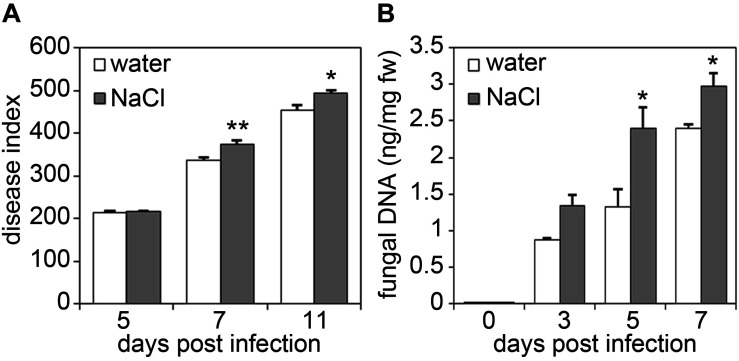
Salt-pre-treated plants are hyper-susceptible to infection with the necrotrophic fungus *Alternaria brassicicola*. Five-weeks-old plants were either watered or treated with 150 mM NaCl solution for 4 days and two leaves per plant were subsequently infected with each six 5 μl droplets of a spore suspension of 1 × 10^5^ spores/ml of *Alternaria brassicicola*. **(A)** Disease indices were calculated 5, 7, and 11 days post infection. **(B)** Fungal biomass was determined by real-time quantitative PCR at indicated days after *Alternaria* infection. *Alternaria* 5.8S rRNA levels are shown relative to the level of the *Arabidopsis* chloroplast-encoded reference gene *Rubisco large subunit*. Shown are mean values with SD (*n* ≥ 18). Significant differences to the water-treated controls are indicated by an asterisk (*p < 0.05, **p < 0.01; Student’s *t*-test). The experiments were each repeated four times with similar results.

To test whether altered pathogen growth rates on salt-stressed plants are also observed upon infection with necrotrophic pathogens, plants were infected with the necrotrophic fungal pathogen *Alternaria brassicicola* (Figure 2). Spore inoculation of salt-treated plants resulted in a significantly enhanced disease index in salt-stressed plants, indicating stronger disease symptom development than in control plants (Figure 2A). Moreover, quantification of fungal DNA in plant leaf tissue confirmed a stronger accumulation of fungal biomass in salt-stressed plants in comparison to that observed in control plants (Figure 2B).

**FIGURE 3:**
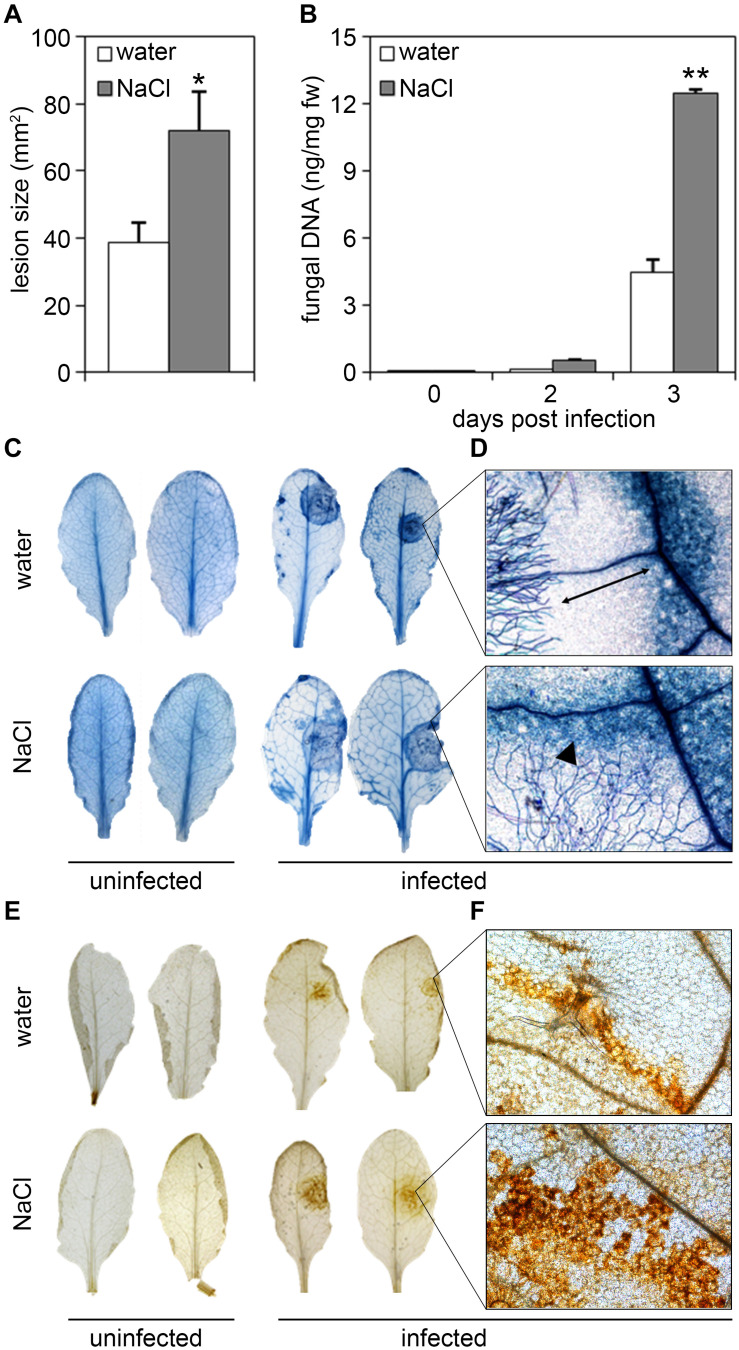
Salt pre-treatment results in decreased resistance toward infection with *Botrytis cinerea*. Five-weeks-old plants were infected with the necrotrophic fungus *Botrytis cinerea* after a 4 days salt-pre-treatment with 150 mM NaCl, control plants were treated with the same amount of water. For the infection, 5 μl spore suspension of 5 × 10^5^ spores/ml was drop-inoculated on one leaf half of the leaf; two leaves per plant were infected. The plants were analyzed for symptom development after 2 and 3 days post infection (dpi). **(A)** Measurement of the lesion size 3 dpi. **(B)** Fungal biomass at day 2 and 3 post-inoculation was determined by RT-qPCR using *Botrytis Actin* genomic DNA levels relative to the level of the *Arabidopsis* chloroplast-encoded reference gene *Rubisco large subunit*. Shown are means and SD (**A**, *n* = 40; **B**, *n* ≥ 18). Significant differences to the water-treated controls are indicated by asterisks (*p < 0.05, **p < 0.01; Student’s *t*-test). **(C)** Trypan blue stain showing visible symptoms after 3 dpi. **(D)** Microscopic analysis of fungal hyphae and dead plant cells at the infection site visualized by Trypan blue stain at 3 dpi. Leaves were analyzed by light microscopy. **(E)** 3,3′-diaminobenzidine tetrahydrochloride (DAB) staining of H_2_O_2_ production after 2 dpi. **(F)** Microscopic analysis of ROS accumulation after DAB staining at 2 dpi. All experiments shown were repeated at least three times with similar results.

Likewise, plants treated with salt prior to infection proved to be more susceptible toward the necrotrophic fungus, *Botrytis cinerea*. Lesion sizes in salt-treated plants were significantly larger than those in untreated plants, as also observed by trypan blue staining of dead plant tissue (Figures 3A,C). Again, fungal growth as determined by quantification of fungal DNA was massively increased in plants pre-treated with salt (Figure 3B). Microscopic examination of trypan-blue-stained infection sites in salt-stressed plants revealed that fungal hyphae spread into the cell death zone (visible as dark blue ring upon trypan blue staining), whereas hyphae in control plants had a more compact appearance (Figure 3D). Formations of lesions was accompanied by the production of reactive oxygen species (ROS) as visualized by staining with 3,3′-diaminobenzidine tetrahydrochloride (DAB). Whereas in control plants ROS accumulation was restricted within the cell death zone (Figure 3E) which was often clearly visible as dark brown circle (Figure 3F), DAB staining produced a stronger and more spreading signal in salt-treated plants (Figures 3E,F). In summary, salt-exposure renders *Arabidopsis* plants more susceptible to infection with both bacterial and fungal pathogens.

### Induction of PTI Responses Is Not Affected by Salt Pre-treatment

**FIGURE 4:**
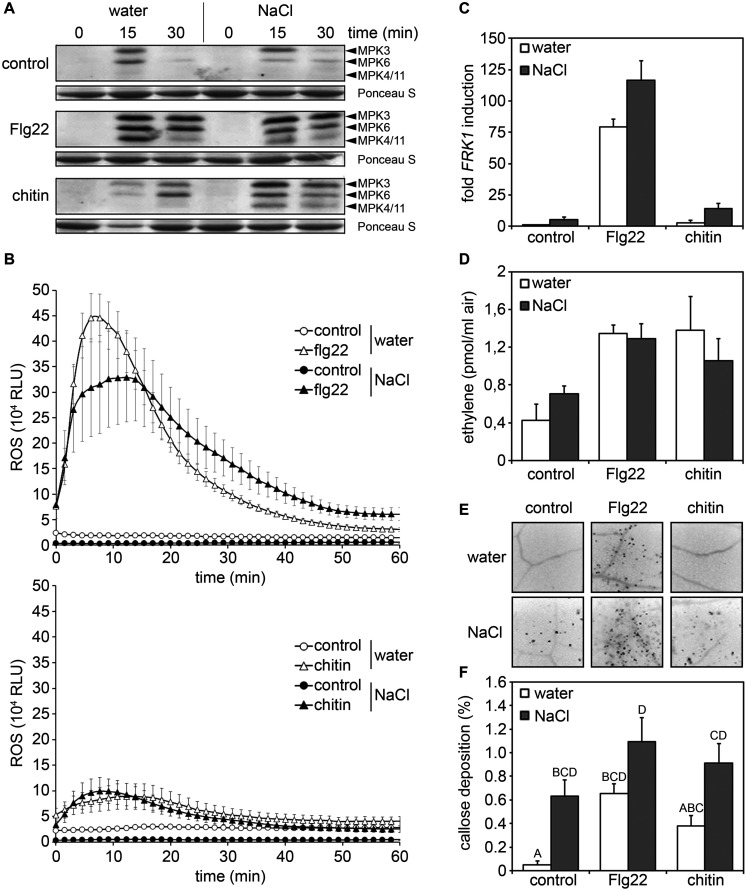
Pattern-triggered immune (PTI) responses are not affected by salt treatment. After a 4 days salt (150 mM NaCl) or water treatment leaves of 5 weeks old plants were treated with 1 μM flg22 or 100 μg/ml chitin or water as control treatment. **(A)** Activated MAPKs in total protein extracts of infiltrated leaves were detected after 15 or 30 min by immunoblotting with the anti-phospho p44/p42 antibody. Ponceau S Red staining served as a loading control. **(B)** Leaf disks were treated with 1 μM flg22 or 100μg/ml chitin, or water as the control and reactive oxygen species (ROS) production was monitored over time. Data present means ± SE (*n* ≥ 6) of relative fluorescence units (RLU). **(C)** Transcriptional profiling of the defense-related gene *FRK1* by RT-qPCR 6 h after treatment by leaf infiltration. Gene expression was normalized to the levels of *EF-1α* transcript and is presented as fold induction compared to the water control. Error bars indicate SD (*n* = 3). **(D)** Ethylene accumulation was measured 4 h after treatment with water (control), 1 μM flg22 or 100 μg/ml chitin. Bars present average values ± SD (*n* ≥ 3). **(E,F)** Callose deposition was determined 24 h post infiltration by staining with aniline blue. Leaf tissues were examined with the microscope under UV-light. Shown are 10-fold enlargements of leaf tissues **(E)** or a quantification of light spots indicating callose depositions **(F)**. Percentages indicate the proportion of fluorescent pixels (callose) compared to the total amount of pixels determined for each digital picture. Shown are means ± SD (*n* ≥ 20). Letters indicate homogenous groups according to *post hoc* comparisons following one-way ANOVA multiple comparison analysis (Dunn test at a probability level of *p* < 0.05). No significant differences to the respective water-treated were detected in panels **(A–D)** (Student’s *t*-test). All experiments were repeated at least two times with similar results.

Plant *R* genes conferring race-cultivar-specific immunity to infection by necrotrophic pathogens have not been described (Veloso and van Kan, 2018), suggesting that immunity to necrotrophic fungal infection is unlikely to be mediated by effector-triggered immunity. Therefore, we tested whether enhanced susceptibility to *Pto*, *Alternaria*, and *Botrytis* infections in salt-treated plants was due to an impaired ability of salt-stressed plants to mount inducible basal defense responses (PTI-associated defense). Toward this end, we analyzed early inducible PTI responses (MAPK activation and ROS accumulation), medium PTI responses (*FRK1* gene expression and ethylene production) as well as a late PTI response (callose apposition). As shown in Figure 4, after application of bacterial flg22 or fungal chitin, mock-treated and salt-treated plants responded with indistinguishable cellular responses such as inducible MAPK activation, ROS production, up-regulation of *FRK1* gene expression and ethylene accumulation. Although salt-stress alone already resulted in increased callose apposition, the amount of pattern-inducible callose depositions was not significantly different in control and salt-stressed plants (Figures 4E,F). Altogether, our findings suggest that decreased immunity to pathogen infection is unlikely to be caused by impaired PTI responses due to salt stress.

### Pre-treatment With Salt Impinges on Phytohormone Levels

**FIGURE 5:**
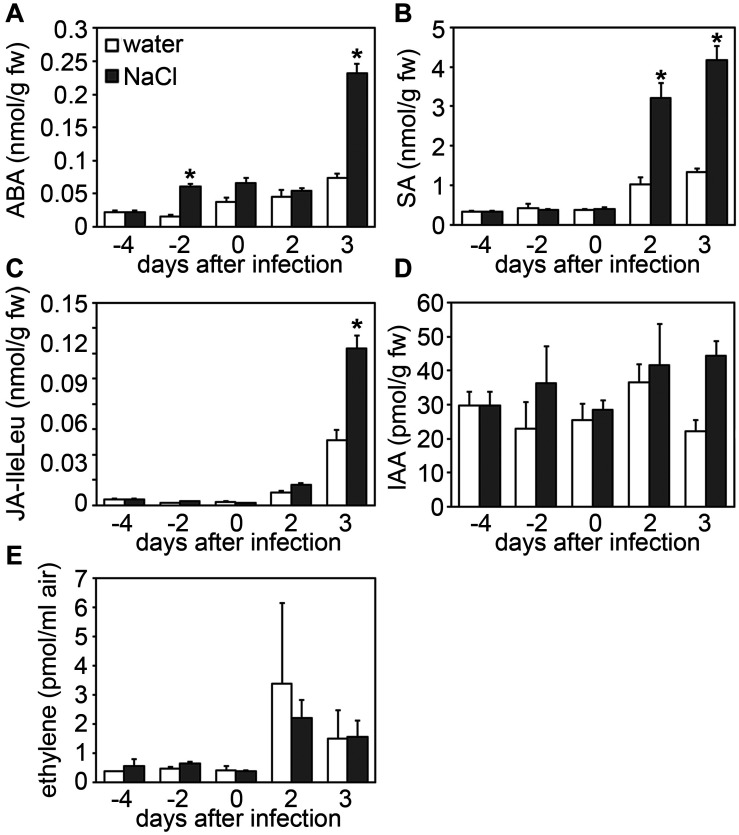
Salt stress results in increased *Botrytis*-induced phytohormone levels. Five-weeks-old plants were either normally watered or treated with 150 mM NaCl at day –4, and on day 0 (equivalent to 4 days salt-treatment) infected with *Botrytis* spores for another 2 or 3 days. At indicated time points samples were collected and phytohormone levels were determined for **(A)** abscisic acid (ABA), **(B)** salicylic acid (SA), **(C)** jasmonic acid (JA-IleLeu), **(D)** indole-3-acetic acid (IAA), and (**E**) ethylene. Shown are means and standard deviation (*n* ≥ 20 for **(A–D)** and *n* = 3 for **E**). Significant differences to the respective water-treated controls are indicated by asterisks (*p < 0.05; Student’s *t*-test). All experiments were repeated at least two times with similar results.

**FIGURE 6:**
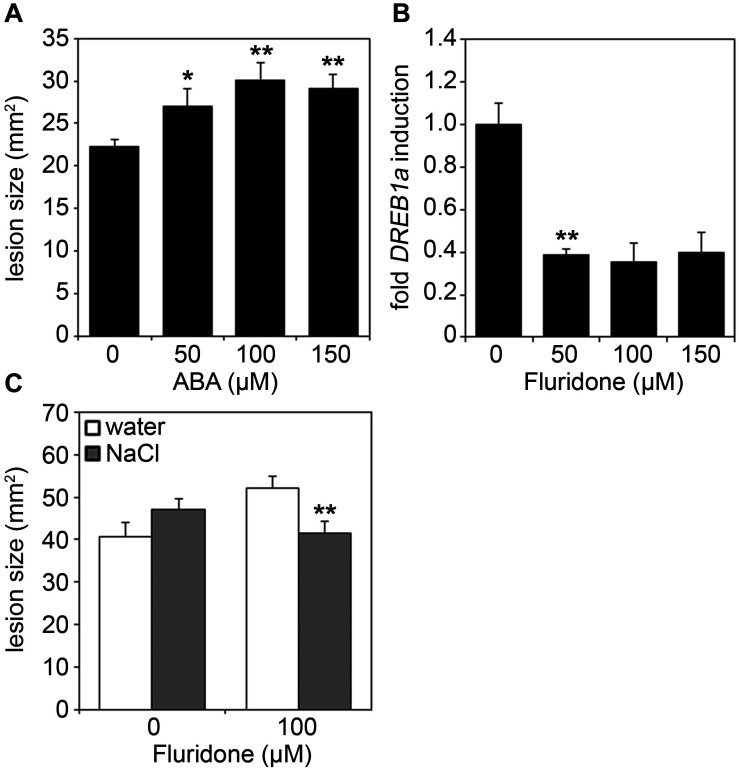
Increased susceptibility to *Botrytis* infection is mediated by ABA. **(A)** Five-weeks-old *Arabidopsis* plants were sprayed with the indicated concentrations of ABA. After 6 h, leaves were drop-inoculated with 5 μl spore suspension of 5 × 10^5^ spores/ml of *B. cinerea*. Lesion sizes were measured 3 days after infection. Shown are means and SD (*n* = 25). Significant differences to the untreated control are indicated by asterisks (*p < 0.05, **p < 0.01; Student’s *t*-test). **(B)** Five-weeks-old *Arabidopsis* plants were sprayed with the indicated concentrations of Fluridone. After 24 h, a 3 h drought stress was applied and samples were analyzed via RT-qPCR for *DREB1a* transcript levels using gene-specific primers. Gene expression was normalized to the levels of *EF-1α* transcript and is presented as fold induction compared to the untreated control which was set to 1. Error bars indicate SD (*n* = 3), significant differences are shown by asterisks (**p < 0.01; Student’s *t*-test). **(C)** Plants were treated for 24 h with or without 100 μM Fluridone, before being further normally watered or treated with 150 mM NaCl. After an additional 4 days, leaves were infected with *B. cinerea* spores and analyzed for lesion development as described in **(A)**. The experiments were repeated with similar results.

During stress adaptation, the homeostasis of phytohormones, such as salicylic acid (SA), jasmonic acid-isoleucine (JA-Ile), abscisic acid (ABA), auxins (e.g., indole-3-acetic acid, IAA) and ethylene, is important for the outcome of cellular responses (Robert-Seilaniantz et al., 2011; Pieterse et al., 2012; Egamberdieva et al., 2017; Ku et al., 2018). To determine whether changes in hormone concentrations may account for the altered immune status of salt-stressed plants, we next analyzed hormone levels in salt and/or *Botrytis*-treated leaf samples. Cellular levels of SA and JA-Ile were found to be more strongly elevated upon *Botrytis* infection combined with prior salt-treatment as compared to fungal infection only, whereas auxin and ethylene levels remained unaffected (Figure 5). Interestingly, ABA levels were not only elevated after combined stress treatment, but already after a 2 days salt treatment prior to infection (Figure 5A). As ABA is known to regulate plant responses to both biotic and abiotic stresses (Kumar et al., 2019; Chen et al., 2020), we next tested whether salt-induced ABA-levels may be causal for the increased susceptibility of salted plants toward fungal pathogens. When spraying ABA onto *Arabidopsis* leaves, we observed bigger lesions after *Botrytis* infection in an ABA concentration-dependent manner (Figure 6A). Next, we pre-treated plants with Fluridone, an inhibitor of phytoene desaturase, an enzyme that converts phytoene to phytofluene in carotenoid biosynthesis (Bartels and Watson, 1978). As carotenoids are the main precursors of ABA in plants (Chen et al., 2020), the inhibition of carotenogenesis also prevents ABA biosynthesis (Toh et al., 2008). Consequently, ABA-mediated drought-induced transcript accumulation of *DREB1a* was abolished upon Fluridone treatment (Figure 6B). Most importantly, Fluridone-treatment also resulted in a loss of salt-stress-induced hyper-susceptibility to infection with *B. cinerea* (Figure 6C), indicating that salt-stress most likely exerts its negative impact on plant immunity through ABA accumulation. To rule out that ABA has a direct positive impact on *B. cinerea* growth, the fungus was grown on medium supplemented with either NaCl, ABA or Fluridone. However, rather than a growth-promoting effect we could observe a growth inhibition caused by all supplements (Supplementary Figure S3).

In conclusion, we report that external ABA application mimics the effect of salt stress by enhancing susceptibility to infection with necrotrophic fungi, while pre-treatment with Fluridone alleviates the reinforcing effect of salt treatment on the plant’s susceptibility to *Botrytis* infection. Thus, we propose that salt stress exerts its effect by a transient increase in ABA levels resulting in hyper-susceptibility toward subsequent attacks by both biotrophic and necrotrophic phytopathogens. Our findings identify ABA as a plant disease-promoting hormone and a factor that governs stress response hierarchy in *Arabidopsis* plants.

## Discussion

Plants constantly have to adjust to fluctuating environmental conditions including both abiotic stress factors and numerous potentially harmful microbes. Evidence accumulates that plant disease resistance is fundamentally influenced by abiotic factors such as light, temperature, water availability or nutrients. Moreover, soil salinity is one of the major abiotic stresses affecting agriculture and was reported to impinge on the plant immune system (Suzuki et al., 2014; Zhang and Sonnewald, 2017; Velasquez et al., 2018; Saijo and Loo, 2020). On one hand, beneficial effects of salt treatment on plant defense were observed, and in former times farmers deliberately salted their lands to protect crops against pathogen infection (Elmer, 1992). On the other hand, salinity was shown to increase susceptibility in certain crops to particular pathogens as exemplified by tomato and chrysanthemum plants which are less resistant toward *Phytophthora* infection when grown under saline conditions (Dileo et al., 2010). Thus, plant genotypes, the type of (abiotic and biotic) stress combinations and application modes may condition the plant’s ability to acclimatize to combined stresses. We observed that upon salt stress, *Arabidopsis* plants are impaired in their resistance to hemibiotrophic bacteria as well as to necrotrophic fungal pathogens (Figures 1–3). This is somewhat unexpected as conditions that are favorable for necrotrophic pathogens are often disadvantageous to biotrophs and vice versa (Glazebrook, 2005), a classical antagonism mediated by the plant hormones SA and JA (Robert-Seilaniantz et al., 2011; Pieterse et al., 2012). However, examples do exist when immunity to both biotrophic and necrotrophic pathogens are similarly affected. For example, *ARABIDOPSIS HISTIDINE KINASE 5* (*AHK5*) mutants exhibit increased susceptibility toward infection with *Pto* DC3000 and *Botrytis* (Pham et al., 2012). Interestingly, *ahk5* genotypes also display an impaired initial stomatal closure during bacterial infection (Desikan et al., 2008). Being natural openings on the leaf surface, stomata are considered one of the main entry points for pathogens (Elad, 1988; Melotto et al., 2017). Hence, defects in stomatal regulation may be a reason for increased pathogen invasion. Moreover, AHK5 is involved in salinity tolerance and *ahk5* mutants better resist salt application compared to the wild type (Pham et al., 2012). Depletion of the CHITIN ELICITOR RECEPTOR KINASE 1 (CERK1), which is essential for the perception of fungal chitin (Miya et al., 2007; Wan et al., 2008), also renders plants more susceptible to salt treatment (Espinoza et al., 2017), indicating that biotic and abiotic stress crosstalk may be mediated by shared signaling components such as AHK5 or CERK1 receptor kinases.

### Cell Wall Damage—Mediator of Cross Talk Between Salt-Stress and Plant Immunity?

The plant cell wall forms an important physical barrier to restrict pathogen invasion. Salt effects on plant immunity may be due to alterations in cell wall integrity. For instance, high salinity causes softening and remodeling of the cell wall (Kesten et al., 2017; Feng et al., 2018), likely by modifying cellulose-pectin crosslinking (Feng et al., 2016; van Zelm et al., 2020). Notably, impairment of cellulose synthesis in *Arabidopsis* results in an increased resistance toward infection with multiple bacterial and fungal pathogens, whereas impairment of pectin biosynthesis causes the opposite phenotype (Bacete et al., 2018).

Cell wall damage inflicted by either abiotic stress or pathogen invasion might be directly perceived by PRRs. Cell wall fragments such as cellulose-derived oligomers or pectin-derived oligogalacturonides (OGs) act as DAMPs to trigger defense responses in an PRR-dependent manner (Gust et al., 2017; Hou et al., 2019). Alternatively, disturbance of cell wall integrity during pathogen invasion can result in the release of phytocytokines, such as plant elicitor peptides (PEPs) and rapid alkalinization factors (RALFs) (Gust et al., 2017; Hou et al., 2019). Notably, PEPs and RALFs are also released during salt stress (Nakaminami et al., 2018; Zhao et al., 2018; Saijo and Loo, 2020), suggesting a DAMP-mediated cross talk between pathogen and salt stress. This hypothesis is further supported by the fact that the corresponding DAMP receptors are also known to be involved in salt stress tolerance. RALF peptides are recognized by the malectin-like receptor kinase FERONIA (FER) and were initially implicated in the regulation of cell expansion and immunity (Haruta et al., 2014; Stegmann et al., 2017). During salt stress, RALF peptides bind both cell wall-localized leucine-rich repeat extensins (LRX) and FER and induce subsequent FER internalization (Zhao et al., 2018). Hence, in concert with extensins, FER may indirectly perceive salinity-induced changes in the cell wall structure. Moreover, FER is not only a RALF receptor but was shown to directly bind to pectin, thus possibly detecting a decreased crosslinking of pectin during stress-induced cell wall softening (Feng et al., 2018). Apart from RALF peptides, pathogen infection, salt stress and cell wall damage all induce the expression of plant elicitor peptides such as AtPep1 and AtPep3, which are perceived by the PEP RECEPTOR 1 (PEPR1) and PEPR2 (Bartels and Boller, 2015; Engelsdorf et al., 2018; Nakaminami et al., 2018), again suggesting DAMP receptor-mediated cross talk between the cellular responses during high salinity and immunity.

Although basal PTI responses such as callose deposition or defense gene expression were indeed partially increased upon salt pre-treatment, enhanced inducibility of these responses upon subsequent microbial pattern treatment was not observed (Figure 4). Moreover, priming usually leads to an enhanced resistance to infection, whereas we rather observed enhanced susceptibility in salt-treated *Arabidopsis* plants to bacterial and fungal infection (Figures 1–3). Thus, although the generation of DAMPs upon high salinity conditions may occur, DAMP-mediated priming of *Arabidopsis* basal immunity (PTI) would be insufficient to protect against microbial infection.

### ABA—A Plant Disease-Promoting Factor and Key Regulator of Plant Stress Hierarchy

Phytohormones are crucial for coordinating cellular responses both to optimal and stressful environmental conditions (Robert-Seilaniantz et al., 2011; Pieterse et al., 2012; Egamberdieva et al., 2017; Ku et al., 2018). In salt-exposed plants subsequently infected with *Botrytis* spores, we observed an increase in the stress-related phytohormones SA, JA-Ile, and ABA (Figure 5). Generally, SA is involved in activating defense mechanisms against biotrophic pathogens, whereas JA-Ile is effective against necrotrophs (Robert-Seilaniantz et al., 2011; Pieterse et al., 2012). However, it was shown that SA signaling is required for resistance toward infection with both bacterial pathogens, such as *Pto* (Delaney et al., 1994), and fungal pathogens, such as *Botrytis* (Ferrari et al., 2003). We observed that plants pre-treated with salt, despite accumulating more SA after fungal infection, are more susceptible against both the hemibiotrophic bacterium *Pto* DC3000 and the necrotrophic fungi *B. cinerea* and *A. brassicicola* (Figures 1–3), making SA an unlikely cause for this phenotype. For the same reason, we consider enhanced levels of JA-Ile and auxin in plants pre-treated with salt prior to *Botrytis*-infection not to be causal for increased susceptibility as these two phytohormones generally correlate with increased plant resistance to infection with necrotrophic fungi (Thomma et al., 1998; Llorente et al., 2008).

Phytohormone crosstalk enables fine-tuning of immune responses and helps the plant to prioritize one defense pathway over the other, dependent on the sequence and type of stresses encountered (Pieterse et al., 2012). Hence, timing, sequence of signaling initiation and hormone concentrations determine the final defense output. We observed that in salt-exposed plants ABA levels, in contrast to SA and JA-Ile levels, were already increased prior to *Botrytis* infection, possibly leading to a pre-disposition of the plant to further infection. In several reports it was demonstrated that ABA negatively impinges on plant immunity toward diverse classes of pathogens. For instance, exogenous application of ABA to monocot and dicot plants enhanced susceptibility toward subsequent infection with pathogenic and beneficial microbes, respectively, as diverse as bacteria (including *Pto*), oomycetes and fungi (including *B. cinerea*), whereas colonization was reduced in ABA biosynthesis or signaling mutants in *Arabidopsis* and tomato (Audenaert et al., 2002; Mohr and Cahill, 2003; Anderson et al., 2004; Thaler and Bostock, 2004; Asselbergh et al., 2007; de Torres-Zabala et al., 2007; Fan et al., 2009; Curvers et al., 2010; Jiang et al., 2010; Pye et al., 2013; Peskan-Berghöfer et al., 2015). In tomato, exogenous administration of ABA could substitute for salt stress and significantly enhance pathogen colonization and disease development. ABA-deficient tomato mutants lacked the predisposition response, which could be restored by complementation of the mutant with exogenous ABA (Dileo et al., 2010). Importantly, plant and even leaf age also have an impact on plant performance upon combined abiotic and biotic stresses. For instance, ABA-mediated impairment of immunity only occurs in older *Arabidopsis* leaves but not in younger rosette leaves (Berens et al., 2019).

## Conclusion

In conclusion, we report that salinity causes elevated ABA levels which in turn negatively affect *Arabidopsis* immunity to infection with (hemi)biotrophic and necrotrophic pathogens. Salt-induced elevated ABA concentrations might subsequently orchestrate changes in gene expression that ultimately result in prioritization of salt stress tolerance over activation of plant immunity in *Arabidopsis.* Moreover, salt-induced perturbations in the composition of root or phyllosphere microbiomes may also impact plant performance upon simultaneous stress exposure (Berens et al., 2019).

## Data Availability Statement

The raw data supporting the conclusions of this article will be made available by the authors, without undue reservation, to any qualified researcher.

## Author Contributions

EH, AAG, and TN conceived and designed the experiments. EH, TI, KF, and BL performed the experiments and analyzed data. IF, MS, AAG, and TN analyzed the data. AAG and TN wrote and revised the article with the input from all co-authors. All authors contributed to the article and approved the submitted version.

## Conflict of Interest

The authors declare that the research was conducted in the absence of any commercial or financial relationships that could be construed as a potential conflict of interest.
