# Supplementary material for: ABA-Dependent Salt Stress Tolerance Attenuates Botrytis Immunity in Arabidopsis
Source: Front Plant Sci. 2020 Nov 17;11:594827. doi: 10.3389/fpls.2020.594827 (PMC7704454; doi:10.3389/fpls.2020.594827)
Supplement: Supplementary file 1 [file Data_Sheet_1.PDF]

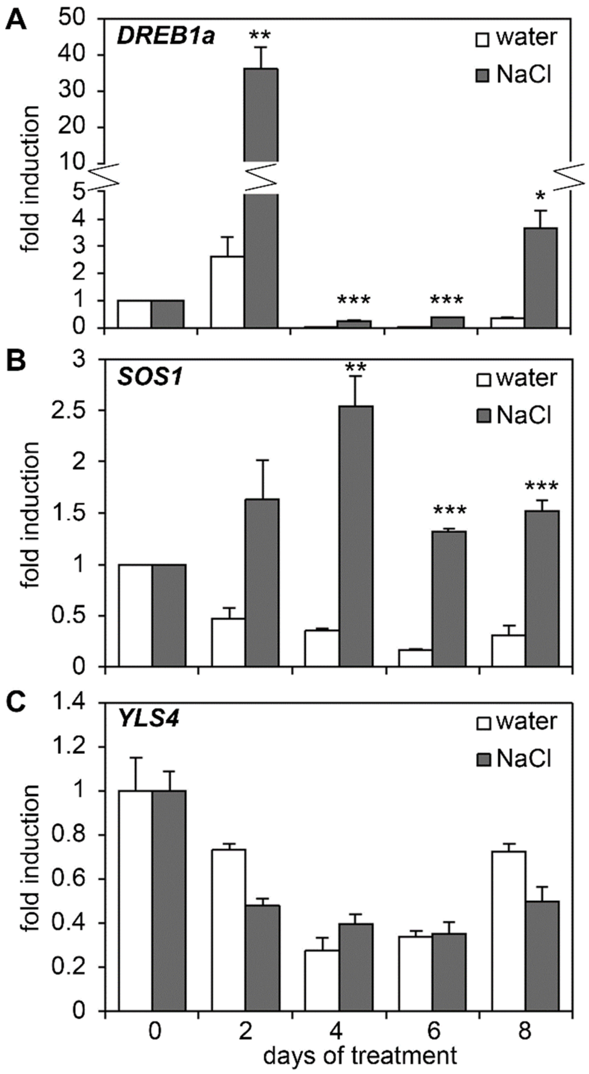

Supplementary Figure S1. Salt-stress is not associated with premature senescence.
5-week-old plants were either normally watered or treated with 150 mM NaCl-solution for the indicated times and transcriptional levels for the salt-induced genes (**A**) *DREB1a* and (**B**) *SOS*, or (**C**) the senescence-associated gene YSL4 were determined in total RNA via RT-qPCR with gene-specific primers. Gene expression was normalized to the levels of *EF-1α* transcript and is presented as fold induction compared to respective samples at time point 0. Error bars indicate standard deviation (n = 3), significant differences are shown by asterisks (* p < 0.05, ** p < 0.01, *** p < 0.001; Student's *t*-test). Shown is one out of two experiments with similar results.
