# Supplementary material for: ABA-Dependent Salt Stress Tolerance Attenuates Botrytis Immunity in Arabidopsis
Source: Front Plant Sci. 2020 Nov 17;11:594827. doi: 10.3389/fpls.2020.594827 (PMC7704454; doi:10.3389/fpls.2020.594827)
Supplement: Supplementary file 2 [file Data_Sheet_2.PDF]

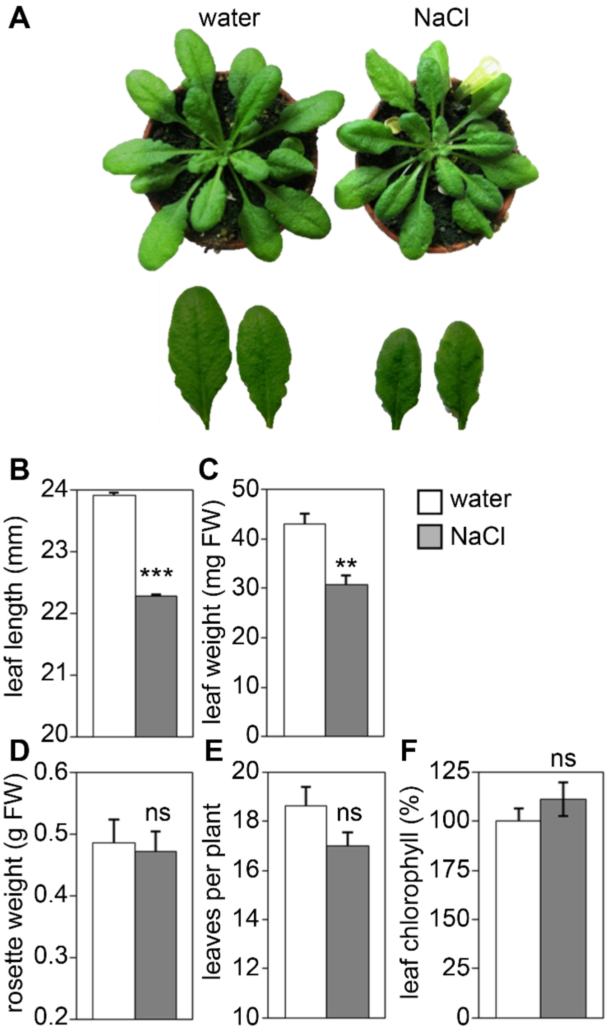

Supplementary Figure S2. Salt-stressed plants show various physiological responses.
(A) 5-week-old plants were either normally watered or treated with 150 mM NaCl-solution and pictures of whole rosettes or single leaves were taken after 4 days. (**B-C)** At day 4 after salt-treatment leaf length (**B**, n = 155) and fresh weight (**C**, n > 120, FW) were determined. Error bars indicate standard deviation, significant differences to the water-control are shown by asterisks (** $p < 0.01$, *** $p < 0.001$; Student's *t*-test). (**D-F)** 4 days after normal watering or salt-treatment the rosette fresh weight (**D**, FW**)**, the amount of leaves per plant (**E**) and the content of leaf chlorophyll (**F**) were determined. Error bars indicate standard deviation (n > 10), but no significant (ns) differences to the water-control were observed (Student's *t*-test).
