# Supplementary material for: ABA-Dependent Salt Stress Tolerance Attenuates Botrytis Immunity in Arabidopsis
Source: Front Plant Sci. 2020 Nov 17;11:594827. doi: 10.3389/fpls.2020.594827 (PMC7704454; doi:10.3389/fpls.2020.594827)
Supplement: Supplementary file 3 [file Data_Sheet_3.PDF]

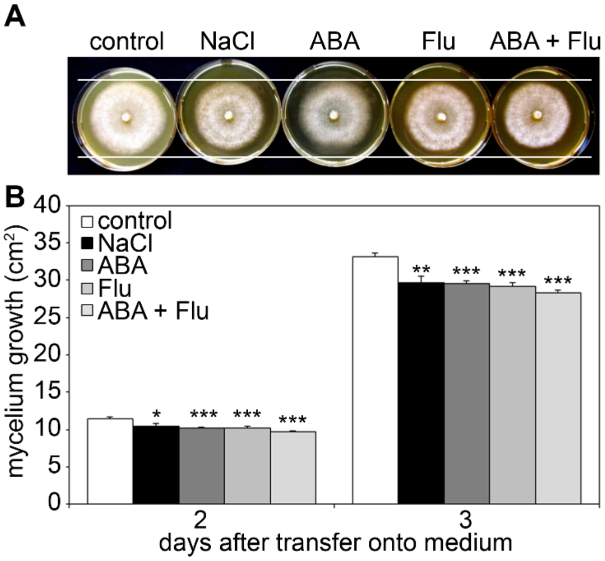

Supplementary Figure S3. Salt-stress and ABA do not increase *B. cinerea* growth on culture medium.

(A) Agar pieces of 5 mm diameter containing *B. cinerea* mycelium were transferred onto potato dextrose agar plates supplemented with 150 mM NaCl, 100 µM ABA, 100µM Fluridone (Flu), or a combination of ABA and Fluridone. *Botrytis* mycelium growth was monitored on day 3 after transfer to the culture plates, white lines indicate outgrowth of the control culture. (**B)** Mycelium proliferation was quantified on days 2 and 3 after transfer to the culture plates. Error bars indicate standard deviation (n = 10), significant differences compared to the untreated control are shown by asterisks (* $p < 0.05$, ** $p < 0.01$, *** $p < 0.001$; Student's *t*-test). The experiment was repeated with similar results.
