# Supplementary material for: ABA-Dependent Salt Stress Tolerance Attenuates Botrytis Immunity in Arabidopsis
Source: Front Plant Sci. 2020 Nov 17;11:594827. doi: 10.3389/fpls.2020.594827 (PMC7704454; doi:10.3389/fpls.2020.594827)
Supplement: Supplementary file 4 [file Data_Sheet_4.PDF]

Supplementary Table S1: Primers used in qPCR analyses

Gene name	Primer name	Primer sequence (5' – 3')
At1g07920/30/40 (EF1α)	eF1a_qF	GAGGCAGACTGTTGCAGTCG
	eF1a_qR	TCACTTCGCACCCTTCTTGA
At2g01980 (SOS1)	SOS1_qF	TGCTGAAGGCATTCTCGACAGTG
	SOS1_qR	ACTGCGCCCCTCAAACCAGA
At2g19190 (FRK1)	FRK1_qF	AGCGGTCAGATTTCAACAGT
	FRK1_qR	AAGACTATAAACATCACTCT
At4g25480 (DREB1a)	DREB1a_qF	CACGGCGGAACAGAGCGAAAA
	DREB1a_qR	CTGTACGGACGGAAGCGGCAAA
At5g11520 (YLS4)	YLS4_qF	CCGGTGGTTCTGTTTTCTCTC
	YLS4_qR	TCAGCTTAACTGGGCTAGGATC
Atcg00490 (Rubisco)	AtRubisco-QF	GCAAGTGTTGGGTTCAAAGCTGGTG
	AtRubisco-QR	CCAGGTTGAGGAGTTACTCGGAATGCTG
Alternaria 5.8S rRNA	Ab_5.8S_qF	ACAATATGAAAGCGGGCTGG
	Ab_5.8S_qR	AAGACGCCCAACACCAAGCA
Botrytis Actin	Bc_actin_qF	CCTCACGCCATTGCTCGTGT
	Bc_actin_qR	TTTCACGCTCGGCAGTGGTGG
